# A case-control study on risk factors of breast cancer in Han Chinese women

**DOI:** 10.18632/oncotarget.21743

**Published:** 2017-10-09

**Authors:** Li-Yuan Liu, Fei Wang, Shu-De Cui, Fu-Guo Tian, Zhi-Min Fan, Cui-Zhi Geng, Xu-Chen Cao, Zhen-Lin Yang, Xiang Wang, Hong Liang, Shu Wang, Hong-Chuan Jiang, Xue-Ning Duan, Hai-Bo Wang, Guo-Lou Li, Qi-Tang Wang, Jian-Guo Zhang, Feng Jin, Jin-Hai Tang, Liang Li, Shi-Guang Zhu, Wen-Shu Zuo, Li-Xiang Yu, Yu-Juan Xiang, Fei Zhou, Liang Li, Qiang Zhang, Qin-Ye Fu, Zhong-Bing Ma, De-Zong Gao, Yu-Yang Li, Lu Liu, Chun-Miao Ye, Yong-Jiu Wang, Wen-Zhong Zhou, Zhi-Gang Yu

**Affiliations:** ^1^ Department of Breast Surgery, The Second Hospital of Shandong University, Jinan, Shandong, 250033, China; ^2^ Department of Breast Surgery, Affiliated Tumor Hospital of Zhengzhou University, Zhengzhou, Henan, 450008, China; ^3^ Department of Breast Surgery, Shanxi Cancer Hospital, Taiyuan, Shanxi, 030013, China; ^4^ Department of Breast Surgery, The First Hospital of Jilin University, Changchun, Jilin, 130021,China; ^5^ Breast Center, The Fourth Hospital of Hebei Medical University, Shijiazhuang, Hebei, 050011, China; ^6^ Department of Breast Surgery, Tianjin Medical University Cancer Institute and Hospital, Tianjin, 300060, China; ^7^ Department of Thyroid and Breast Surgery, The First Affiliated Hospital of Binzhou Medical University, Binzhou, Shandong, 256603,China; ^8^ Department of Breast Surgery, Cancer Hospital, Chinese Academy of Medical Sciences, Beijing, 100021, China; ^9^ Department of General Surgery, Linyi People’s Hospital, Linyi, Shandong, 276003, China; ^10^ Breast Disease Center, Peking University People's Hospital, Beijing, 100044, China; ^11^ Department of General Surgery, Beijing Chaoyang Hospital, Beijing, 100043, China; ^12^ Breast Disease Center, Peking University First Hospital, Beijing, 100034, China; ^13^ Breast Center, Qingdao University Affiliated Hospital, Qingdao, Shandong, 266003, China; ^14^ Department of Breast and Thyroid Surgery, Weifang Traditional Chinese Hospital, Weifang, Shandong, 261041, China; ^15^ Department of Breast Surgery, The Second Affiliated Hospital of Qingdao Medical College, Qingdao Central Hospital, Qingdao, Shandong, 266042, China; ^16^ Department of General Surgery, The Second Affiliated Hospital of Harbin Medical University, Harbin, Heilongjiang, 150001, China; ^17^ Department of Breast Surgery, The First Affiliated Hospital of China Medical University, Shenyang, Liaoning, 110001, China; ^18^ Department of General Surgery, Nanjing Medical University Affiliated Cancer Hospital, Cancer Institute of Jiangsu Province, Nanjing, Jiangsu, 210009, China; ^19^ Department of Breast and Thyroid Surgery, Zibo Central Hospital, Zibo, Shandong, 255036, China; ^20^ Department of Breast Surgery, Yantai Yuhuangding Hospital, Yantai, Shandong, 264000, China; ^21^ Breast Cancer Center, Shandong Cancer Hospital, Jinan, Shandong, 250117, China

**Keywords:** breast cancer, Han Chinese, risk factors, case-control study

## Abstract

This study aimed to investigate risk factors associated with breast cancer among Han Chinese women in northern and eastern China. A matched case-control study involving 1489 patients with breast cancer and 1489 controls was conducted across 21 hospitals in 11 provinces in China, from April 2012 to April 2013. We developed a structured questionnaire to record information from face-to-face interviews with participants. Student’s t-tests, Pearson’s chi-square tests, and univariate and multivariate conditional logistic regression analyses were used to identify variables with significant differences between the case and control groups. Ten variables were identified (P*<*0.05): location, economic status, waist-to-hip ratio, menopause, family history of breast cancer, present life satisfaction, sleep satisfaction, milk products, behavior prevention scores, and awareness of breast cancer. We identified a comprehensive range of factors related to breast cancer, among which several manageable factors may contribute to breast cancer prevention. Further prospective studies concerning psychological interventions, sleep regulation, health guidance, and physical exercise are required. A screening model for high-risk populations should be put on the agenda.

## INTRODUCTION

Breast cancer is the most common type of cancer worldwide; the incidence is continuing to rise, and it is the leading cause of cancer-related death among women [1, 2]. World Health Organization (WHO) statistics show there were 1.67 million new breast cancer cases diagnosed in 2012, accounting for 25% of all cancers diagnosed that year [3]. Reports in China indicate the annual increase in the incidence of breast cancer has doubled or tripled over the past two decades, making it the leading cancer among women [4–6].

Characteristics of established risk factors for breast cancer may vary among countries. Better understanding the characteristics of local risk factors may inform more effective breast cancer prevention strategies [7]. When risk factors are well understood, healthcare providers are able to supply women with more accurate information regarding their individual risk of developing breast cancer [8]. Cancer risk assessment has emerged as an important component of cancer risk counseling [9–11].

Worldwide, numerous studies have sought understand the risk factors for breast cancer. However, there has been no consensus because of differences in sample sizes, races that comprised study populations, and local customs. Most epidemiological studies have evaluated risk factors for breast cancer based on large sample sizes in Western populations. However, these risk factors are not based on Chinese women and cannot be directly applied in China, because risk factors may differ across different populations [12–14]. In China, breast cancer risk factors have received considerable attention. Several case-control studies have been conducted to screen potential risk factors in various local areas; however, most studies included small sample sizes. Currently, national monitoring data on risk factors among the Chinese general population are limited. This study aimed to investigate risk factors for breast cancer among Han Chinese women. Risk factors determined in our study will help to identify Chinese women who have an increased risk of breast cancer, and support effective early detection and disease prevention interventions.

## RESULTS

**Figure 1:**
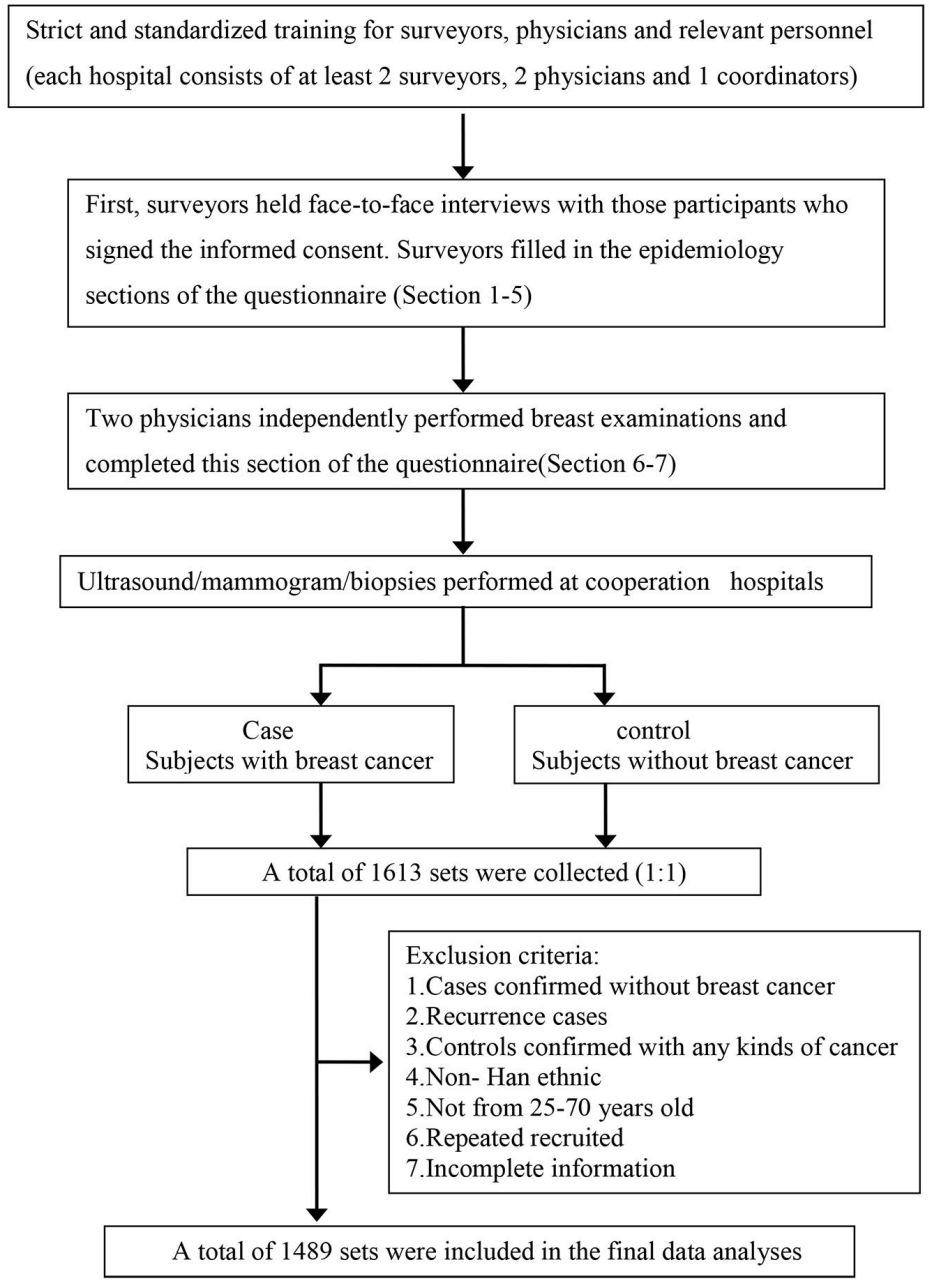
Flow chart of the study implementation process

Figure 1 shows the study implementation process. We initially recruited 1613 pairs of 1:1 matched cases and controls. Of these women, 1489 pairs were eligible for enrollment in the study, as 124 pairs were excluded after logical checks (16 with benign diseases in the case group, 46 with malignant diseases in the control group, 10 with non-Han ethnicity, seven with non-matched age, 13 with duplicate enrollment, 22 with relapse diseases, and 18 with incomplete information). We found that 1120 participants (37.61%) had full understanding of the questionnaire, 1450 (48.69%) mostly understood the questionnaire, 224 (7.52%) had partial understanding, and eight (0.27%) did not understand the questionnaire. In total, 1714 women (57.56%) fully cooperated with the investigation, 1035 women (34.75%) were basically cooperative, and 41 women (1.37%) did not cooperate.

Among the 1489 patients with breast cancer, there were 1128 cases with invasive ductal carcinoma (accounting for 75.8% of the study population), 127 (8.5%) with intraductal carcinoma, 24 (1.6%) with invasive lobular carcinoma, and 194 (10.7%) with other types of cancer (including mucinous breast carcinoma, neuroendocrine carcinoma, comedocarcinoma and medullary carcinoma). Luminal A type, luminal B type, HER-2 type, and triple negative types accounted for 10.7% (n=159), 49.9% (n=743), 8% (n=119), and 8.5% (n=126) of cases, respectively. In addition, 322 cases were estrogen receptor negative (21.63%), 1018 were estrogen receptor positive (68.37%), 149 had lost estrogen receptor status (10.01%), 417 were progesterone receptor negative (28.01%), 951 were progesterone receptor positive (63.09%), and 121 had lost progesterone receptor status (8.13%).

Demographic characteristics for the case and control groups are shown in Table 1. There were statistically significant differences between the two groups. Of the 1489 cases, 92 (6.2%) were aged 25–34 years, 451 (30.3%) were aged 35–44 years, 588 (39.5%) were aged 45–54 years, 315 (21.2%) were aged 55–64 years, and 43 (2.9%) were aged over 65 years. Patients aged over 45 years accounted for 63.5% of all cases, and there were no significant differences between the two groups (χ^2^=5.172, P=0.222). However, there were differences in education levels (χ^2^=65.333, P<0.001), location (χ^2^=60.900, P<0.001), family average revenue (χ^2^=98.827, P<0.001), economic status (χ^2^=104.593, P<0.001), social status (χ^2^=77.895, P<0.001), and awareness of breast cancer (χ^2^=20.585, P<0.001) between the two groups.

**Table 1:** Basic demographic information for the case and control groups

Variable	Case N(%)	Control N(%)	*χ*^*2*^	*P*
**Location**				
Urban	704 (49.1)	907 (63.6)	60.90	<0.001
Rural	729 (50.9)	519 (36.4)		
**Age**			5.712	0.222
25-	92 (6.2)	113 (7.6)		
35-	451 (30.3)	486 (32.6)		
45-	588 (39.5)	568 (38.1)		
55-	315 (21.2)	283 (19.0)		
65-	43 (2.9)	39 (2.6)		
Education			65.333	<0.001
Elementary or low	279 (19.4)	187 (13.0)		
Middle	485 (33.8)	402 (28.1)		
High	443 (30.9)	471 (32.9)		
College	219 (15.3)	346 (24.1)		
Postgraduate	9 (0.6)	27 (1.9)		
**Family average revenue(RMB)**			98.827	<0.001
<1000	108 (7.5)	32 (2.2)		
1000-1999	243 (16.8)	151 (10.5)		
2000-2999	400 (27.7)	342 (23.7)		
3000-4999	351 (24.3)	440 (30.5)		
≥5000	344 (23.8)	477 (33.1)		
**Economic status**			104.593	<0.001
High	38 (2.6)	56 (3.8)		
Good	313 (21.5)	510 (35.0)		
Average	915 (62.8)	812 (55.7)		
Poor	190 (13.1)	80 (5.4)		
**Social status**			77.895	<0.001
High	43 (3.0)	62 (4.3)		
Good	327 (22.7)	516 (35.7)		
Average	989 (68.6)	813 (56.3)		
Poor	82 (5.7)	53 (3.7)		
**Awareness of breast cancer**			20.585	<0.001
Highly aware	248 (16.7)	347 (23.3)		
Poorly aware	1241 (83.3)	1142 (76.7)		

There were no differences between the case and control groups in age at menarche (7−11 years, 74.0% vs. 73.5%), menstrual pattern (irregular, 6.4% vs. 5.7%), and marital status (never married, 6.4% vs. 4.9%). However, there were significant differences between the two groups in postmenopausal status (χ^2^=8.244, P=0.004) and number of births (χ^2^=36.026, P<0.001). No significant differences were found for breastfeeding, number of miscarriages, and use of oral contraceptives (Table 2).

**Table 2:** Comparison of reproductive and menstrual characteristics between the case and control groups

Variable	Case N(%)	Control N(%)	*χ*^*2*^	*P*
**Age at menarche (years)**			0.655	0.721
7--11	1076 (74.0)	1074 (73.5)		
12--13	361 (24.8)	366 (25.0)		
≥14	17 (1.2)	22 (1.5)		
**Menstrual pattern**			0.467	0.494
Regular	1279 (93.6)	1264 (94.3)		
Irregular	87(6.4)	77 (5.7)		
**Menopause**			8.244	0.004
Yes	499 (34.6)	423 (29.6)		
No	942 (65.4)	1005 (70.4)		
**Marriage**			3.053	0.081
Ever	1394 (93.6)	1416 (95.1)		
Never	95 (6.4)	73 (4.9)		
**Oral contraceptives**				
Yes	109 (7.7)	107 (7.5)	0.032	0.858
NO	1302 (92.3)	1311 (92.5)		
**Number of births**				
0	34 (2.3)	43 (2.9)		
1--2	1222 (83.3)	1312 (89.6)	36.026	<0.001
≥3	211 (14.3)	110 (7.5)		
**Number of miscarriages**				
0	554 (40.4)	575 (41.7)		
1--2	680 (49.6)	688 (49.9)	2.171	0.338
≥3	136 (9.9)	115 (8.3)		
**Breastfeeding**			0.629	0.428
Yes	1303 (91.3)	1311 (92.1)		
No	124 (8.7)	112 (7.9)		

Table 3 shows the characteristics of chronic diseases in the case and control groups. There were statistically significant differences in hypertension (χ^2^=4.625, P=0.032), benign tumor of the breast (χ^2^=26.957, P<0.001), galactophore hyperplasia (χ^2^=14.520, P<0.001), nipple discharge (χ^2^=5.849, P=0.016), and family history of breast cancer (χ^2^=13.168, P<0.001). Variables not associated with significant differences were diabetes mellitus (4.0% vs. 3.4%), inverted nipple (1.3% vs. 0.8%), and multiple breasts (1.3% vs. 2.0%).

**Table 3:** Comparison of chronic diseases between the case and control groups

Variable	Case N(%)	Control N(%)	*χ*^*2*^	*P*
**Hypertension**			4.625	0.032
Yes	189 (12.8)	152 (10.3)		
No	1285 (87.2)	1325 (89.7)		
**Diabetes mellitus**			0.791	0.374
Yes	59 (4.0)	50 (3.4)		
No	1410 (96.0)	1422 (96.6)		
**Benign tumor of breast**			26.957	<0.001
Yes	84 (5.9)	166 (11.3)		
No	1339 (94.1)	1298 (88.7)		
**Galactophore Hyperplasia**			14.52	<0.001
Yes	252 (17.3)	336 (22.9)		
No	1207 (82.7)	1130 (77.1)		
**Spillage of nipple**			5.849	0.016
Yes	19 (1.3)	37 (2.5)		
No	1433 (98.7)	1419 (97.5)		
**Inverted nipple**			2.167	0.141
Yes	19 (1.3)	11 (0.8)		
No	1432 (98.7)	1442 (99.2)		
**Multiple breasts**			2.070	0.150
Yes	19 (1.3)	29 (2.0)		
No	1430 (98.7)	1427 (98.0)		
**Family history of breast cancer**			13.168	<0.001
Yes	99 (6.7)	55 (3.7)		
No	1382 (93.3)	1423 (96.3)		
**Family history of first-degree relatives**			3.479	0.062
Yes	57 (4.0)	39 (2.7)		
No	1375 (96.0)	1392 (97.3)		
**Family history of second-degree relatives**			8.619	0.003
Yes	43 (3.0)	20 (1.4)		
No	1384 (97.0)	1409 (98.6)		

There were significant differences between the case and control groups in cigarette smoking (χ^2^=5.862, P=0.015), tea drinking (χ^2^=5.250, P=0.022), and sleep satisfaction (χ^2^=15.892, P<0.001), but no differences in alcohol drinking (1.0% vs. 0.8%), coffee drinking (4.8% vs. 5.6%), and physical activity (71.6% vs. 74.1%) (Table 4). Characteristics of dietary habits in the case and control group are shown in Supplementary Table 2.

**Table 4:** Comparison of behavioral habits between the case and control groups

Variable	Case N(%)	Control N(%)	*χ*^*2*^	*P*
**Cigarette smoking**			5.862	0.015
Yes	30 (2.0)	14 (0.9)		
No	1455 (98.0)	1467 (99.1)		
**Second-hand smoking**			15.498	<0.001
Yes	547 (61.3)	457(52.1)		
No	345 (38.7)	421 (47.9)		
**Alcohol drinking**			0.340	0.560
Yes	15 (1.0)	12 (0.8)		
No	1466 (99.0)	1471 (99.2)		
**Tea drinking**			5.250	0.022
Yes	337 (23.0)	388 (26.7)		
No	1127 (77.0)	1066 (73.3)		
**Coffee drinking**			0.801	0.371
Yes	70 (4.8)	80 (5.6)		
No	1383 (95.2)	1360 (94.4)		
**Physical activity**			2.341	0.126
Yes	420 (28.4)	383 (25.9)		
No	1057 (71.6)	1094 (74.1)		
**Sleep satisfaction**			15.829	<0.001
Very satisfied	178 (12.2)	216 (14.9)		
Satisfied	1007 (68.9)	1022(70.3)		
Dissatisfied	249 (17.0)	205 (14.1)		<0.001
Very dissatisfied	27 (1.8)	10 (0.7)		

Body size measures for cases and controls are shown in Table 5. The mean height (± standard deviation) of cases was 160.03 cm (± 4.78 cm) and that of controls was 160.38 cm (± 4.31 cm). Body mass index (BMI) was higher in cases compared with controls (t=2.599, P=0.009). There were statistically significant differences in waist circumference (t=5.106, P=0.009), hip circumference (t=2.176, P=0.030), and waist-to-hip ratio (WHR) (t=2.704, P=0.007) between the case and control groups.

**Table 5:** Comparison of body size measures between the case and control groups

Variable	Case	Control	*t/χ*^*2*^	*P*
**Height(cm)**	160.03±4.78	160.38±4.31	-2.003	0.045
**Weight(kg)**	62.32±9.17	61.74±8.31	1.736	0.078
**BMI (kg/m**^2^**)**	24.33±3.44	24.01±3.12	2.599	0.009
***Distribution***				
<24.0	687 (49.2)	717 (51.1)	10.887	0.004
24.0–28.0	520 (37.2)	552 (39.3)		
>28.0	190(13.6)	135 (9.6)		
**Waist circumference(cm)**	2.40±0.29	2.35±0.25	5.106	<0.001
**Hip circumference(cm)**	2.84±0.34	2.81±0.36	2.176	0.030
**WHR**	0.85±0.08	0.84±0.07	2.704	0.007
***Distribution***				
<0.85	535 (44.4)	633 (49.3)	6.180	0.013
≥0.85	671 (55.6)	650 (50.7)		

Table 6 shows blood parameters for the case and control groups. No significant differences between the groups were observed in adiponectin, including total adiponectin (t=−1.393, P=0.164) and high-molecular-weight (HMW) adiponectin (t=−0.840, P=0.401). In addition, there were no significant differences in triglyceride (t=1.580, P=0.144) and total cholesterol (t=0.093, P=0.926) levels.

**Table 6:** Comparison of blood parameters between the case and control groups

Variable	Case	Control	*t*	*P*
	X ±SD	X±SD		
**Total adiponectin (μg/ml)**	6.353±3.551	6.563±3.721	-1.393	0.164
**HMW adiponectin**	2.517±1.885	2.583±1.876	-0.840	0.401
**Glucose**	5.294±1.206	5.202±1.258	1.986	0.047
**Triglyceride**	1.314±0.958	1.253±0.806	1.580	0.144
**Total cholesterol**	4.761±0.981	4.757±1.033	0.093	0.926

All variables included in the questionnaire were analyzed using matched conditioned logistic regression analysis (Table 7). Significant differences (α=0.05) between the case and control groups were observed for: location, education, economic status, social status, hypertension, family history of breast cancer, menopause, BMI, WHR, sleep satisfaction, present life satisfaction, cigarette smoking, bean products, vegetables , milk products, behavior prevention scores, and awareness of breast cancer. Multivariate Cox regression models were performed to analyze risk factors for breast cancer (α=0.10). Nine factors were significantly related to breast cancer, for which the odds ratios (OR) and 95% confidence intervals (CI) were: location, 1.269 (0.984–1.638, P=0.067); economic status, 1.237 (1.019–1.501, P=0.032); family history of breast cancer, 2.418 (1.361–4.294, P=0.003); menopause, 1.982 (1.360–2.888, P<0.001); WHR, 1.329 (0.983–1.797, P=0.065); sleep satisfaction, 1.412 (1.140–1.749, P=0.002); present life satisfaction, 1.852 (1.436–2.390, P<0.001); milk products, 0.813 (0.716–0.923, P=0.001); behavior prevention scores, 0.685 (0.517–0.907, P=0.008); and awareness of breast cancer, 0.675 (0.520–0.876, P=0.003).

**Table 7:** Logistic regression analysis of breast cancer-related factors

Variable	*OR*	*Unadjusted OR* *95% CI*		*P*	*OR*	*Adjusted OR* *95% CI*		*P*
		*Lower*	*upper*			*Lower*	*upper*	
Location	1.923	1.627	2.273	0.000	1.269	0.984	1.638	0.067
Education	0.698	0.640	0.760	0.000	-	-	-	-
Economic status	1.854	1.637	2.100	0.000	1.237	1.019	1.501	0.032
Social status	1.743	1.523	1.995	0.000	NA	NA	NA	NA
Hypertension	1.287	1.015	1.633	0.037	-	-	-	-
Family history of breast cancer	1.880	1.334	2.649	0.000	2.418	1.361	4.294	0.003
Menopause	1.725	1.333	2.234	0.000	1.982	1.360	2.888	0.000
BMI	1.134	1.010	1.273	0.034	NA	NA	NA	NA
WHR	1.408	1.115	1.780	0.004	1.329	0.983	1.797	0.065
Sleep satisfaction	1.310	1.141	1.505	0.000	1.412	1.140	1.749	0.002
Present life satisfaction	1.951	1.660	2.292	0.000	1.852	1.436	2.390	0.000
Cigarette smoking	2.143	1.136	4.041	0.019	-	-	-	-
Bean products	1.109	1.006	1.222	0.037	-	-	-	-
Vegetables	1.170	1.047	1.307	0.006	NA	NA	NA	NA
Milk products	0.879	0.836	0.925	0.000	0.813	0.716	0.923	0.001
Behavioral prevention score	0.780	0.744	0.818	0.000	0.685	0.517	0.907	0.008
Awareness of breast cancer	0.617	0.506	0.753	0.000	0.675	0.520	0.876	0.003
Family history of breast cancer ^*^ Present life satisfaction					1.545	1.159	2.060	0.003
WHR^*^ Present life satisfaction					1.342	1.189	1.515	0.000
WHR^*^ Sleep satisfaction					1.064	1.009	1.121	0.022

-, no data; NA, not available, there were strong correlations between these factors and other factors, and NA means that it was not included in the multivariate analysis; CI, confidence interval; BMI, body mass index; WHR, waist-to-hip ratio.

Multiplicative model interaction was assessed with a cross-product interaction term in our multivariate logistic regression model. Two-factor interaction analyses were conducted among statistically significant variables selected by the multivariate analysis. Positive interactions (at α=0.05) were observed for: family history and present life satisfaction; WHR and present life satisfaction; and WHR and sleep satisfaction (Table 7). It is important to note that the interaction obtained through the logistic regression analysis represents a multiplicative model. For example, the interaction between family history and present life satisfaction indicates that for females with a family history of breast cancer, those with poorer life satisfaction have an increased breast cancer risk.

## DISCUSSION

Development of breast cancer is a complicated and continuous progress, characterized by multi-step, multi-factor, and environment-gene interactions in origin. Although many studies on breast cancer development have been conducted, reported results varied widely. This may be related to disparities in study designs, geographical features, and lifestyle and healthcare factors. It is important to investigate and clarify risk factors for breast cancer, especially manageable factors, with which better prevention strategies could be formulated.

We described a case-control study involving 2978 Chinese Han women. In total, 75.8% of breast cancer cases were diagnosed as invasive ductal carcinoma, which is consistent with national and international reports. In China, invasive ductal carcinoma accounts for about 70% of all female breast cancers, whereas other tumor types (e.g., invasive lobular carcinoma) account for no more than 5% [15–18]. In our study, 50% of breast cancer cases were luminal B type, which is a much higher rate than in previous reports (11–23%). This disparity may be attributable to the new classification standard published by the St Gallen International Expert Consensus [19], which included both the progesterone receptor positive range (20%) and ki67 cutoff value (14%) for classification. According to this classification standard, some cases originally recognized as luminal A type were reclassified as luminal B type.

**Figure 2:**
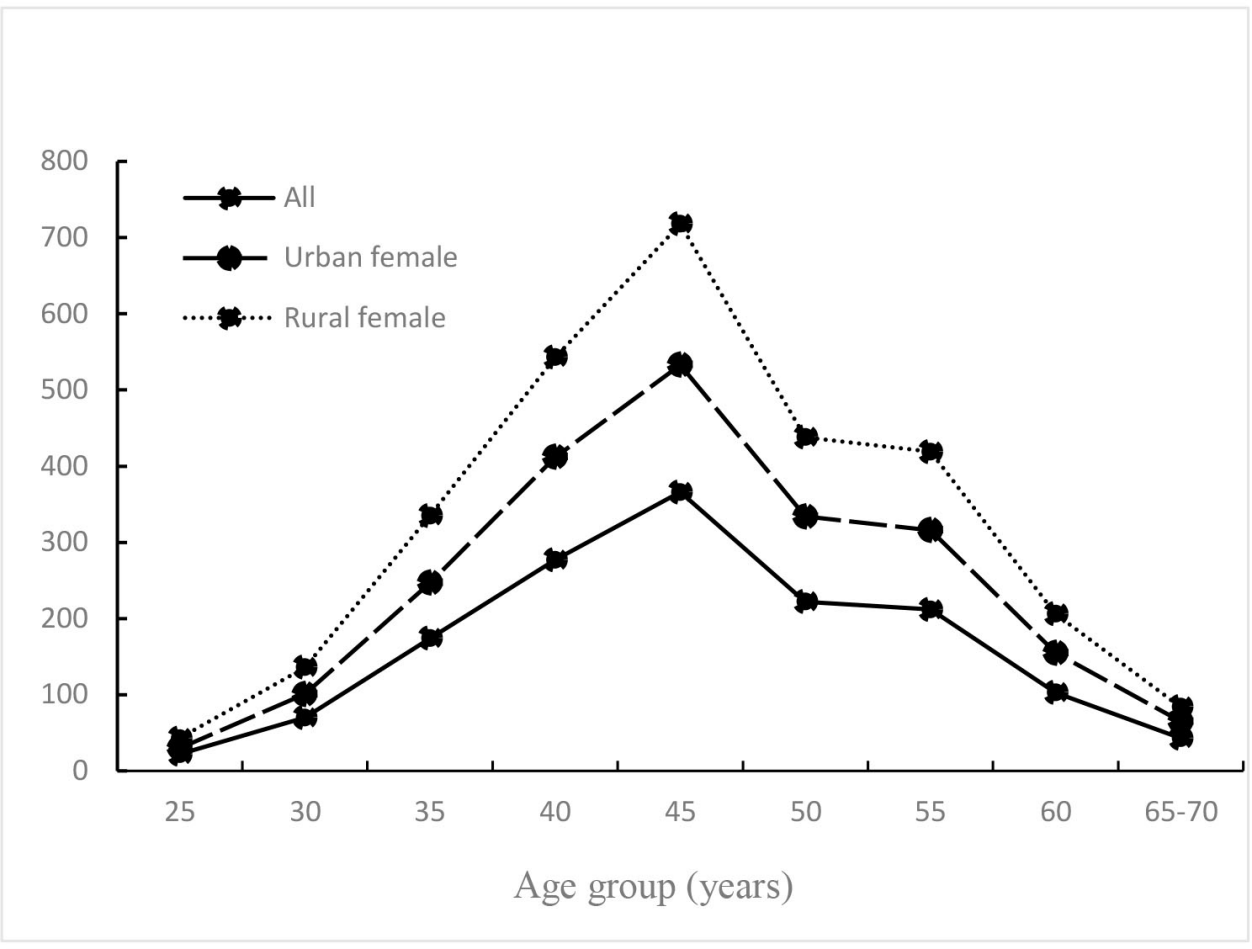
Distribution of breast cancer cases by age group

Our study (Figure 2) showed that the peak incidence of breast cancer was around age 45–55 years in both rural and urban areas. This is about 10 years earlier than in American and other Western countries (age 65 years). Compared with our previous study [20], that found bimodal patterns of incidence (one at 55–60 years and another at 60–65 years), no such patterns were observed. Previous Chinese studies reported obvious bimodal patterns of age-specific incidence, with the incidence of premenopausal breast cancer reported to be much higher than the postmenopausal incidence. However, this pattern changed over the past several years. For example, in the Shanghai Female Study [21] involving females aged 35–80 years, the age-specific incidence of breast cancer presented a gradual upward trend from 1973. Two age peaks were revealed before 2002 (especially 1998–2002), whereas a gradual shift toward a unimodal peak was observed from 2003–2007, which is consistent with our study.

Previous studies demonstrated a genetic susceptibility to breast cancer. Females with a family history of breast cancer, especially among first-degree relatives, were more likely to develop breast cancer. Moreover, the risk was further increased in cases where more than one breast cancer case had been diagnosed among first-degree relatives [22, 23]. In our study, family history, first-degree relative family history, and second-degree relative family history were researched, and multivariate logistic regression and OR assessment were performed. We found that a family history of breast cancer doubled the risk of developing the disease (OR=2.418), which showed a similar trend to our previous study (OR=7.08) [24] and another Western report [23].

Obesity is another factor that contributes to the increasing incidence of breast cancer [25–27]. The incidence of overweight and obesity among female adults increased from 29.8% in 1983 to 38.0% in 2013 [28]. Currently, BMI and WHR are the most common measures for defining obesity and investigating associations between obesity and breast cancer. Compared with BMI, WHR may provide a better mean for evaluating central obesity, which is more common in China. Several studies have shown that high WHR is related to increased breast cancer risk [29, 30]. In our study, both high BMI and WHR were correlated with increased risk of breast cancer (OR 1.010 and 1.115, respectively) in the univariate logistic regression analysis, but only high WHR remained after the multivariate logistic regression analysis (OR 1.329). This is consistent with results reported by Ali Montazeri [31] and Pathak [32]. However, the mechanisms by which overweight and obesity influence breast cancer development have not yet been elucidated. It has been proposed that high BMI is connected to increased insulin and insulin-like growth factors, which in turn contribute to the elevated risk of breast cancer. Arendt et al. [33] showed that a micro-inflammatory state, increased estrogen levels, and decreased insulin sensitivity secondary to obesity were potential links between obesity and breast cancer. A reasonable diet, physical exercise, medication, and even surgery may facilitate weight control, which may reduce breast cancer risk. Future prospective studies are needed o determine whether such methods would work.

A dietary pattern that includes a high-fat component, soy, dairy products, meat, fruits, and vegetables is supposed to affect breast cancer development and progress, although no consistent conclusions have been reached [34–36]. In our univariate logistic regression analysis, soy and dairy products were related to a reduced risk of breast cancer, with dairy products remaining after the multivariate logistic regression analysis. This is consistent with studies among females in Hong Kong [37]. However, a meta-analysis by Dong et al. [38] revealed no associations between dairy products and breast cancer risk. This disparity may be partly explained by regional variations in eating habits.

Psychological status should not be overlooked as a potential factor related to breast cancer development [39, 40]. Many studies demonstrated that negative life events, depression, anxiety, irritability, and unhealthy psychological factors contributed to the development of system secondary to emotional stress [41, 42]. In our study 12 items were used to assess overall life satisfaction and six items to assess current life satisfaction. High scores indicated low satisfaction or dissatisfaction, whereas low scores indicated high satisfaction. We found that low current life satisfaction was associated with an increased risk of breast cancer (OR=1.852), suggesting that psychological interventions should be considered in breast cancer prevention.

Previous studies showed that poor sleep quality (reported prevalence of 5–40%), was related to elevated risk of a variety of tumors [43–45]. In our study, insomnia, early awakening, sleeping late, and subjective sleep quality were correlated with breast cancer development in the univariate logistic regression analysis. The multivariate logistic regression analysis showed poor sleep quality was associated with increased risk of breast cancer (OR=1.412), which is consistent with some previous reports [46]. Given current epidemiological evidence, there is no agreement about the association between sleep quality and breast cancer, and the potential mechanism needs to be further studied.

We also investigated awareness of and knowledge about breast cancer-related symptoms and risk factors. Only 72.8% of participants knew breast cancer was a common cancer among females; 83.3% reported low awareness, and only 16.7% had high awareness. About 52.7% of women recognized a lump as a clinical manifestation of breast cancer, although only about 30.0% recognized other breast cancer-related symptoms such as breast discomfort, enlarged lymph nodes, nipple inversion, and nipple discharge. In addition, 63.3% knew that family history of breast cancer and long-term use of estrogen-like medicines were risk factors for breast cancer. The rates of awareness of other risk factors were below 30%. Correlation analysis suggested that high awareness was a protective factor for breast cancer, highlighting the importance and necessity of targeted publicity and education programs.

Based on previous findings that obesity may be related to increased breast cancer risk and poorer outcomes, we explored the association between adipokines and breast cancer. Adiponectin is considered the key link between obesity and breast cancer [47], especially postmenopausal breast cancer, although current studies have reported mixed conclusions [48–50]. In our study, both total adiponectin and HMW adiponectin serum levels were tested with the enzyme-linked immunosorbent assay (ELISA) method. When analyzed as continuous numeric variables, no associations were observed. However, when distinguished by a cut-off value on the receiver operating characteristic curve, a high HMW adiponectin level was correlated with reduced breast cancer risk. This conclusion was valid among postmenopausal women. No association between total adiponectin level and breast cancer risk was observed, which is consistent with previous studies [51, 52]. Thus, the serum HMW adiponectin level was more likely to impact breast cancer development than the total adiponectin level.

Our study was a retrospective case-control study. As women self-reported their parity, breastfeeding, disease, and alcohol use histories, our findings may be subject to recall bias. To minimize recall bias, several similar questions were asked in different sections of the questionnaire. A 1:1 matched case-control design (by age and hospital) was used to control for possible confounders, and all interviewers were required to complete standardized training. In future, we aim to validate the risk and protective factors identified in this study using a case-cohort study.

We identified a comprehensive range of factors related to breast cancer. Among these there were several manageable factors that may contribute to breast cancer prevention. Future prospective studies are needed that consider psychological interventions, sleep regulation, health guidance, and physical exercise. In addition, a screening model for high-risk populations should be put on the agenda.

## MATERIALS AND METHODS

We conducted a multi-center, hospital-based, case-control study of breast cancer among women in northern and eastern China. This study was funded by the Ministry of Health of the People’s Republic of China, and took place in 21 hospitals located in 11 provinces, from April 2012 to April 2013.

### Study population

The target population was female outpatients with breast cancer aged 25–70 years in 21 hospitals. Cases and controls were matched (1:1) on age (± 3 years), diagnosis hospital (same hospital), and timing of examination (within 2 months). Inclusion criteria for breast cancer cases were: (1) newly diagnosed and histologically confirmed breast cancer; (2) Han ethnic group; and (3) females aged 25–70 years. Exclusion criteria for patients with breast cancer were: recurrent or metastatic breast cancer, complication of other malignant tumors by clinical or pathological diagnosis, and <25 or >70 years of age. Inclusion criteria for the control group were: (1) negative physical examination results; (2) negative ultrasound scans of breast and/or mammographic screening results; (3) no evidence of cancer or history of cancer; and (4) Han ethnic group. Patients who had a neoplastic disease at any other site, or history of cancer or other major chronic disease were excluded from the study. Data collection strictly adhered to the inclusion and exclusion criteria. After excluding those with inadequate information or missing data, 1489 case-control pairs were involved in this study.

### Data collection

We developed a self-designed structured questionnaire to record information obtained from participants during face-to-face interviews. The interview questionnaire was based on: published articles; the Gail, Claus, and international models; and discussions with experts in breast surgery, epidemiology, statistics, nutrition, and molecular biology. To minimize recall bias, several similar questions were asked in different sections of the questionnaire. A preliminary investigation was performed to assess the practicality and effectiveness of the survey. After repeated revisions, the final interviewer-administered questionnaire comprised seven parts. (1) Demographic characteristics and female physiological and reproductive factors (e.g., age, age at menarche, age at menopause, number of miscarriages, breastfeeding, dysmenorrhea, menopausal status). (2) Chronic diseases and family history (e.g., benign breast disease diabetes mellitus, hypertension, and family history of breast cancer—first- and second-degree relatives). (3) Lifestyle habits, including smoking (including passive smoking), alcohol intake, and dietary habits. (4) Medication and chemical exposure history (including hair dyes, antidiabetic agents). (5) Breast cancer-related knowledge (risk factors for breast cancer, early signs and symptoms of breast cancer). (6) Medical records, specifically, information gathered from the clinical breast examination (including results from visual examination, palpation, and related diagnostic tests; histological and immunohistochemical diagnoses of breast cancer patients were also collected). (7) Physical measurements (height, weight, BMI, hip and waist circumference, WHR, blood pressure, blood glucose, triglyceride, and total cholesterol).

For each participant, a 4-ml non-fasting blood sample was collected using an EDTA vacutainer. After sedimentation, each blood sample was stored vertically in a freezer at −80°C. Total and HMW adiponectin levels were assayed from plasma using human total adiponectin and HMW adiponectin quantitative ELISA kits, respectively (RD systems, SRP300, SHWAD0). All analyses were performed at the Central Research Laboratory, the Second Hospital of Shandong University. Testing of fasting plasma glucose, triglyceride, and total cholesterol were performed by the collaborating hospitals’ clinical laboratories.

### Quality control

Interviewers were medical professionals and medical post-graduates. All interviewer candidates were required to complete standardized training and were certified to conduct independent surveys. To minimize recall bias, several similar questions were asked in different sections of the questionnaire; for example, we used date of birth and age (years) to express actual age, years of schooling and highest degree to express education level, number of pregnancies = number of births + number of abortions, number of children = number of boys + number of girls. Solutions to contradictions are shown in Supplementary Table 1. The questionnaires and forms were coded twice, and were double-entered by different clerks. Inconsistent records were manually checked and corrected. Computer programs were used to check the logic and reasonable range of responses throughout the questionnaire to identify contradictory responses.

### Ethics statement

All procedures performed involving human participants were in accordance with the ethical standards of the Second Hospital of Shandong University Research Committee. Written informed consent was obtained from all participants by investigators as part of the interview.

### Statistical analyses

The database was established using Epidata 3.1 software (Epidata Association, Odense, Denmark). Frequencies and percentages were calculated for variables such as demographic characteristics, physiological and reproductive factors, chronic diseases and family history, lifestyle habits, medication and chemical exposure history, breast cancer-related knowledge, medical records, and physical measurements. We used Student’s t-tests and Pearson’s chi-square tests for the univariate analysis, and found 17 variables had significant differences (location, education, economic status, social status, hypertension, family history of breast cancer, menopause, BMI, WHR, sleep satisfaction, present life satisfaction, cigarette smoking, bean products, vegetable, milk products, behavior prevention scores, and awareness of breast cancer). Multivariate conditional logistic regression analyses were used to stratify independent variables with ORs and 95% CIs. All data were analyzed using SPSS version 16.0 (SPSS Inc., Chicago, IL, USA). A two-sided P-value <0.05 was considered to be statistically significant.

## SUPPLEMENTARY MATERIAL TABLES


