# Supplementary material for: A case-control study on risk factors of breast cancer in Han Chinese women
Source: Oncotarget. 2017 Oct 9;8(57):97217–30. doi: 10.18632/oncotarget.21743 (PMC5722557; doi:10.18632/oncotarget.21743)
Supplement: Supplementary file 1 [file oncotarget-08-97217-s001.pdf]

A case-control study on risk factors of breast cancer in Han Chinese women

SUPPLEMENTARY MATERIALS

Supplementary Table 1: Logical judgment principle for selected variables in the questionnaire

Item 1	Item 2	Item 3	Solution
date of birth	years old	—	When in contradiction, item1 to be followed
years of schooling	the highest degree	—	When in contradiction, item2 to be followed
number of pregnancies	number of births	number of abortions	Number of pregnancies = number of births + number of abortions, according to the logical relationship
number of children	number of boys	number of girls	Number of children = number of boys + number of girls, according to the logical relationship

Supplementary Table 2: Comparison of dietary habits between the case and control groups

Variable	Case N(%)	Control N(%)	χ^2	*P*
**Bean products**			4.795	0.187
1	206 (14.1)	222 (15.1)		
2	525 (36.0)	567 (38.4)		
3	568 (39.0)	554 (37.6)		
4	159 (10.9)	132 (8.9)		
**Red meat**			20.618	<0.001
1	335 (23.2)	365 (24.8)		
2	466 (32.3)	568 (38.6)		
3	522 (36.1)	442 (30.0)		
4	121 (8.4)	96 (6.5)		
**Milk products**			50.641	<0.001
1	186 (12.8)	292 (20.0)		
2	332 (22.8)	405 (27.7)		
3	493 (33.9)	432 (29.6)		
4	443 (30.5)	332 (22.7)		
**Corn**			7.205	0.066
1	121 (8.4)	91 (6.2)		
2	366 (25.3)	414 (28.4)		
3	668 (46.2)	660 (45.2)		
4	292 (20.2)	295 (20.2)		
**Fried food**			4.417	0.22
1	64 (4.5)	49 (3.4)		
2	246 (17.2)	278 (19.1)		
3	614 (42.9)	634 (43.7)		
4	508 (35.5)	491 (33.8)		
**Vegetables**			8.757	0.033
1	801 (55.1)	881 (60.4)		
2	367 (25.2)	319 (21.9)		
3	241 (16.6)	214 (14.7)		
4	45 (3.1)	45 (3.1)		
**Garlic**			6.868	0.076
1	198 (13.7)	164 (11.2)		
2	405 (27.9)	441 (30.2)		
3	557 (38.4)	592 (40.5)		
4	290 (20.0)	265 (18.1)		
**Pickled foods**			3.966	0.265
1	220 (15.3)	216 (14.8)		
2	312 (21.7)	294 (20.2)		
3	505 (35.1)	563 (38.7)		
4	400 (27.8)	383 (26.3)		
**Ham**			3.111	0.375
1	35 (2.4)	36 (2.5)		
2	158 (10.9)	190 (13.1)		
3	498 (34.5)	490 (33.7)		
4	752 (52.1)	738 (50.8)		
